# Antibiotic Resistance among Gastrointestinal Bacteria in Broilers: A Review Focused on *Enterococcus* spp. and *Escherichia coli*

**DOI:** 10.3390/ani13081362

**Published:** 2023-04-15

**Authors:** Jessica Ribeiro, Vanessa Silva, Andreia Monteiro, Madalena Vieira-Pinto, Gilberto Igrejas, Filipa S. Reis, Lillian Barros, Patrícia Poeta

**Affiliations:** 1 Microbiology and Antibiotic Resistance Team (MicroART), Department of Veterinary Sciences, University of Trás-os-Montes and Alto Douro (UTAD), 5000-801 Vila Real, Portugal; 2 Associated Laboratory for Green Chemistry (LAQV-REQUIMTE), University NOVA of Lisbon, 2829-516 Lisbon, Portugal; 3 Centro de Investigação de Montanha (CIMO), Instituto Politécnico de Bragança, Campus de Santa Apolónia, 5300-253 Bragança, Portugal; 4 Laboratório Associado para a Sustentabilidade e Tecnologia em Regiões de Montanha (SusTEC), Instituto Politécnico de Bragança, Campus de Santa Apolónia, 5300-253 Bragança, Portugal; 5 Department of Genetics and Biotechnology, University of Trás-os-Montes and Alto Douro (UTAD), 5000-801 Vila Real, Portugal; 6 Functional Genomics and Proteomics Unit, University of Trás-os-Montes and Alto Douro (UTAD), 5000-801 Vila Real, Portugal; 7 Associate Laboratory for Animal and Veterinary Science (AL4AnimalS), University of Trás-os-Montes and Alto Douro (UTAD), 5000-801 Vila Real, Portugal; 8 Department of Veterinary Science, University of Trás-os-Montes and Alto Douro (UTAD), 5000-801 Vila Real, Portugal; 9 Veterinary and Animal Research Centre (CECAV), University of Trás-os-Montes and Alto Douro (UTAD), 5000-801 Vila Real, Portugal

**Keywords:** antibiotic resistance, broilers, food animals, gastrointestinal microbiota, one health

## Abstract

**Simple Summary:**

Chicken meat has become one of the most consumed meats worldwide, and antibiotics have been used to ensure high levels of production. However, antibiotic usage in animal production has contributed to the development of antibiotic-resistant bacteria, largely among intestinal microbiota. *Enterococcus* spp. and *Escherichia coli* are frequently found in the gastrointestinal tract of chickens, and the presence of resistant strains has been revealed by several studies. *Enterococcus* spp. isolated from broilers have shown resistance to at least seven classes of antibiotics, while *E. coli* have shown resistance to at least four. Furthermore, some clonal lineages, such as ST16, ST194, and ST195 in *Enterococcus* spp. and ST117 in *E. coli*, have been identified in broilers and humans. These data suggest that bacteria can be transmitted through the consumption of contaminated animal-source food, direct contact with animals, or environmental exposure. Therefore, the main goal of this review was to highlight the existing literature on the gastrointestinal microbiota in broilers and antibiotic-resistant *Enterococcus* spp. and *E. coli* of broiler origin.

**Abstract:**

Chickens can acquire bacteria at different stages, and bacterial diversity can occur due to production practices, diet, and environment. The changes in consumer trends have led to increased animal production, and chicken meat is one of the most consumed meats. To ensure high levels of production, antimicrobials have been used in livestock for therapeutic purposes, disease prevention, and growth promotion, contributing to the development of antimicrobial resistance across the resident microbiota. *Enterococcus* spp. and *Escherichia coli* are normal inhabitants of the gastrointestinal microbiota of chickens that can develop strains capable of causing a wide range of diseases, i.e., opportunistic pathogens. *Enterococcus* spp. isolated from broilers have shown resistance to at least seven classes of antibiotics, while *E. coli* have shown resistance to at least four. Furthermore, some clonal lineages, such as ST16, ST194, and ST195 in *Enterococcus* spp. and ST117 in *E. coli*, have been identified in humans and animals. These data suggest that consuming contaminated animal-source food, direct contact with animals, or environmental exposure can lead to the transmission of antimicrobial-resistant bacteria. Therefore, this review focused on *Enterococcus* spp. and *E. coli* from the broiler industry to better understand how antibiotic-resistant strains have emerged, which antibiotic-resistant genes are most common, what clonal lineages are shared between broilers and humans, and their impact through a One Health perspective.

## 1. Gastrointestinal Bacteria in Chickens

**Figure 1:**
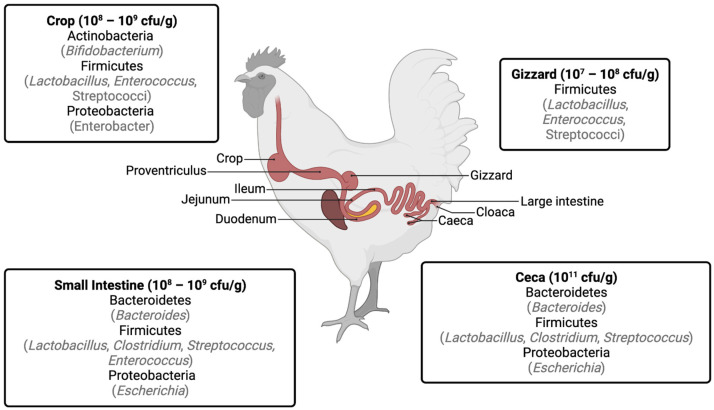
Major bacterial populations and counts (colony-forming unit per gram of sample) in the different sections of the gastrointestinal tract of chickens (in gray are identified the genera of each phylum). Modified from [5]. Information extracted from [7,8,9,10,11,12].

Chickens can acquire bacteria at the embryonic stage during egg formation in the oviduct and transport through the reproductive tract. The hatching environment also plays an important role in the chicken’s microbial profile [1]. When hatching, newborns are exposed to bacteria from the eggshells [2]. Most eggshells are contaminated immediately after eggs are laid, and they are largely contaminated by contact with dirty surfaces [3]. Post-hatch bacterial acquisition diverges for many reasons such as production practices, diet, and environment [4]. The gastrointestinal tract of chickens consists of the crop, stomach (proventriculus and gizzard), small intestine (duodenum, jejunum, ileum), ceca, large intestine, and cloaca, and each has individual metabolic functions that define the microbial community (Figure 1) [5]. The birds’ age also determines the composition and functions of these communities [6]. 

Regardless of the age or section of the gastrointestinal tract, Firmicutes, Proteobacteria, Actinobacteria, and Bacteroidetes are the most prevalent phyla in chickens [9,13]. The crop microbiota includes *Bifidobacterium*, *Klebsiella pneumoniae*, *Klebsiella ozaenae*, *Escherichia coli*, *Escherichia fergusonii*, *Enterobacter aerogenes*, *Eubacterium* spp., *Pseudomonas aeruginosa*, *Micrococcus luteus*, *Staphylococcus lentus*, and *Sarcina* spp. However, it is mainly colonized by Gram-positive bacteria such as *Lactobacillus* spp. [7,8]. The proventriculus and gizzard are predominantly colonized by lactobacilli due to their resistance to acid pH [5,9]. Enterococci can also be found in the proximal parts of the chicken’s digestive tract (crop, gizzard, and proventriculus) [9]. The duodenum and jejunum are colonized at low densities by lactobacilli, enterococci, and *Clostridiaceae*, while the ileum is mostly colonized by *Lactobacillus*, *Clostridiaceae*, *Enterococcus*, and *Streptococcus* [8,11]. Proteobacteria, such as *Escherichia coli*, can also be found in the small intestine [8]. The ceca have the most complex microbial community of the gastrointestinal tract, and it includes Gram-positive cocci, *Clostridium* spp., *E. coli*, *Lactobacillus* spp., *Streptococcus* spp., and *Bacteroides* spp. [8,12]. The composition of the fecal microbiota can differ according to the time chosen for sample collection: if the samples are collected after cecum evacuation, the microbial composition might be identical to the cecal microbiota; if the small intestine digesta passes through the colon right after voiding the cecal excretion, the microbial composition can be a combination of cecal and ileal microbiota; or, if the samples are collected previously to the new cycle of cecal contents voiding to the colon, the microbial composition can be identical to ileal microbiota [14]. Overall, the microbial density and the main phyla in the different sections of the gastrointestinal tract of the chicken are summarized in Figure 1. 

## 2. Development of Antibiotic Resistance by Gastrointestinal Bacteria in Broilers

The growth of the human population, the increase in incomes, and the changes in consumer trends (more protein in the diet) have increased the consumption of animal products. This high demand for animal products led to high levels of animal production [15]. A large sector of the food-producing animal industry is represented by poultry, and over the last three decades, it has expanded with an annual growth rate of over 5%, while the bovine and swine industries grew 1.5% and 3%, respectively [16,17]. The poultry meat production varies, but in Europe, over 80% is chicken [18]. Commercial meat-type chicken production is highly specialized and includes two types of farms: breeding hens to obtain fertile eggs and broilers to obtain meat [19]. According to Statistics Portugal (INE), the gross production of chicken meat in Portugal in 2021 recorded a level similar to the previous year (+0.8%), having reached 313 thousand tons [20]. 

To ensure high levels of production, antimicrobials have been used in animal production for therapeutic purposes, disease prevention, and growth promotion [21]. In 2020, in Portugal, approximately 179.1 tons of antimicrobials were sold to food animal producers, and the antimicrobial classes for veterinary use with higher sales were tetracyclines (34.4%), penicillins (22.1%), macrolides (11.4%), pleuromutilins (7.2%), polymyxins (6.7%), quinolones (4.2%), sulfonamides (4.1%), lincosamides (3.5%), aminoglycosides (2.8%), amphenicols (2.5%), and trimethoprim (1%) [22]. The use of antimicrobials as growth promoters involves administering subtherapeutic concentrations of antibiotics orally or mixed into feed or water with the final goal of increasing the rate of weight gain and the efficiency of the feed [21]. In poultry, antibiotics are generally administered to the entire flock, and antibiotic usage for disease prevention is allowed in all large poultry-producing countries [23,24]. 

Antimicrobial use in livestock is linked to the development of antimicrobial resistance, and antibiotic resistance mechanisms can be easily spread within microbial communities [25]. The development of resistant strains has raised some public health concerns, and to overcome this issue, the EU and South Korea have banned the use of antibiotics as feed additives [26,27]. Other countries, such as Denmark, Japan, and Canada, formally monitor antibiotic use and the development of antimicrobial resistance [28,29,30]. In the US, in early 2017, the Food and Drug Administration (FDA) banned antimicrobials used as growth promoters, but they continue to be legally administered via animal feed for disease prevention, often at lower dosages and for longer periods, similarly to production uses now prohibited [21,31]. However, in some European countries, a substantial decline in the sales of antimicrobials for food-producing animals has been observed [32]. Antibiotic-free poultry production has also become popular in many developed countries, particularly within the US poultry industry, mainly due to a consumer perception that antibiotic-free produced poultry is superior to conventionally raised poultry [33]. However, broilers raised with no antibiotics are more susceptible to enteric diseases that can negatively impact their intestinal health and general welfare [34]. Improving poultry production, increasing poultry immunity, and reducing the spread of disease are vital, and studies have reported that these can be achieved by adding diverse, potentially valuable ingredients to the feed or drinking water of poultry flocks [35]. 

A significant increase in the use of antibiotics is caused by broiler farming, and their permanent use disrupts the gastrointestinal metabolism of chickens [9,36]. The gastrointestinal tract is considered the main intervening part of productivity, pathogen entrance, and disease prevention [37]. Any disturbance might lead to poor digestion or absorption of nutrients or the inability to fight pathogens and the development of a disease [5]. Since antibiotics have been largely used as feed additives in animal production for therapeutic or growth promotion purposes, it is crucial to pay attention to the health of the broiler’s gastrointestinal tract and the development of drug-resistant bacteria [36]. 

Vancomycin is one of the “last-line” antibiotics used to treat life-threatening infections caused by Gram-positive bacteria [38]. Avoparcin, a glycopeptide antibiotic analog of vancomycin, was introduced as a feed additive in chicken feed in 1987, and, even though it was banned from the EU in 1997, the development of vancomycin-resistant Enterococci might have been potentiated by its use as a feed additive in livestock [38,39,40,41]. Furthermore, β-lactams are among the most used classes of antibiotics both in humans and animals, and an increasing trend of gastrointestinal colonization by extended-spectrum β-lactamase (ESBL) producing bacteria has been observed in commercial poultry farms and humans [42,43]. ESBL-producing bacteria are often reported among broilers, and the acquisition of ESBL genes among gastrointestinal microflora may play an important role in the spread of multidrug-resistant bacteria among humans, animals, and the environment via the food chain [44,45,46]. 

Antibiotic-resistant bacteria and antibiotic-resistant genes can be found in many hosts and environments, including wild animals, organically produced food animals, and even in newborn babies never exposed to antibiotics [44,47,48,49]. In addition, both pathogenic and non-pathogenic bacteria can harbor antimicrobial resistance genes [50]. Antimicrobial resistance studies have mainly focused on clinical pathogens, but recently, due to the emergence of zoonotic diseases, the impact of antimicrobial resistance on animals, agricultural practices, wildlife, and the environment has gained new attention and led to cooperation from various sectors [51]. Thus, Commission Implementing Decision 2020/1729 of 17 November 2020 determined that to monitor and report antimicrobial resistance, commensal *Enterococcus faecalis*, *Enterococcus faecium,* and *E. coli*, as well as food-producing animals, such as broilers, must be considered [52].

## 3. *Enterococcus* spp.

*Enterococcus* spp. are Gram-positive, catalase-negative, non-spore-forming, facultative anaerobic lactic acid bacteria that can be found in the gastrointestinal microbiota of humans and other animals [53]. These bacteria can tolerate many adverse conditions, surviving for several months in hostile environments, including extreme pH and temperature conditions (between 10 ℃ and 45 ℃) and high NaCl concentrations [54]. Since they prefer intestinal habitats and are widespread, robust, and easy to cultivate, they are often used as indicators of fecal contamination and integrated hygiene criteria for water and food products [55]. They are also appropriate for veterinary and human resistance surveillance systems [56,57]. These naturally gut-oriented bacteria were considered harmless commensal bacteria, but when the commensal relationship with the host is disturbed, enterococci can cause aggressive infections [58]. They are currently recognized as one of the main nosocomial pathogens and are progressively becoming more resistant to antimicrobial agents. These species have also been associated with an increasing number of hospital-acquired infections in both human and veterinary medicine [59,60,61]. In poultry, *Enterococcus* spp. can cause several diseases such as osteomyelitis, femoral head necrosis, spondylitis, skeletal disease, and arthritis. Furthermore, these organisms have been linked to musculoskeletal disease in broiler breeders and broilers [56,62].

### 3.1. Enterococci Species Diversity

More than 50 different species of enterococci have been described, and *E. faecalis*, *E. faecium*, *Enterococcus hirae*, and *Enterococcus durans* are the most common species in the gastrointestinal tract of chickens [60,63,64]. *E. faecalis* and *E. faecium* are almost entirely responsible for nosocomial enterococcal infections [65]. In poultry, *E. faecalis* is responsible for increased first-week mortality, amyloid arthropathy in layers, and valvular endocarditis, salpingitis, peritonitis and arthritis in broilers, while *E. faecium* has been linked to septicemic disease in white Peking ducklings [66,67]. In 1-day-old chicks, *E. faecalis* and *E. faecium* can be found mainly in the intestines, while *E. durans* can be found in the crop. Later, at an age of 3–4 weeks, *E. durans* can be found in the intestines [68]. A study that included meat samples from turkeys and organic and conventional chickens revealed that *E. durans* was the most common species isolated from conventional chickens [63]. Moreover, a European study performed with commensal enterococci from healthy cattle, pigs, and chickens revealed that *E. durans* was among the most prevalent enterococci [69]. *E. hirae* is the fourth most common *Enterococcus* species identified in poultry, and it has been demonstrated that these bacteria can colonize the small intestines of 3-week-old chickens, and, even though less frequently, 12-week-old chickens [68,70]. In the past few years, *E. hirae* has been among the most common species of opportunistic pathogenic bacteria in poultry and can often be isolated from broiler chickens with endocarditis [71,72,73]. 

Different species can also be identified in particular age groups, such as *Enterococcus cecorum* in older poultry [68]. In fact, an age-dependent succession of enterococcal species colonization seems to occur in chickens. Chickens are initially colonized by *E. faecalis*, but this population is then replaced, mostly by *E. faecium*. This replacement may occur due to the use of tylosin, to which *E. faecium* is frequently resistant, as a growth promoter. When the chickens mature, these species seem to be replaced by *E. cecorum* [68,74]. The earliest existence of commensal *E. cecorum* in the digestive tract of chickens was recognized at the age of 3 to 4 weeks, and by 12 weeks of age, this species was the most prevalent enterococcal component in the crop and intestines of chickens [68,70]. *E. cecorum* was isolated from the cecal flora of chickens and described as a gastrointestinal commensal of various mammals and birds [75]. However, *E. cecorum* is an opportunistic pathogen that may also play a role as an etiological agent of diseases in humans (nosocomial infections), chickens, and racing pigeons [72,76,77,78]. Several studies have described this bacterium as an emerging pathogen in the poultry industry [79,80,81,82]. Borst et al. (2017) identified *E. cecorum* with pathogenic genotypes in one-week-old naturally infected broilers. These authors also reported that the ability to colonize the gastrointestinal tract early in life may offer a competitive advantage to pathogenic *E. cecorum* strains and potentiate dissemination through a flock [83]. *E. cecorum* has been recognized as a cause of inflammatory musculoskeletal lesions, such as enterococcal spondylitis in chickens, broiler breeders, and broilers in Europe, Canada, and the US [62,83,84,85]. 

### 3.2. Antimicrobial Resistance in Enterococci

Enterococci have been described as intrinsically resistant to β-lactam antibiotics, such as penicillins (ampicillin, amoxicillin/clavulanic acid, penicillin G, methicillin, piperacillin), carbapenems (imipenem), and cephalosporins (cefoperazone, ceftriaxone) [86]. In addition, they can also be extrinsically resistant due to the accumulation of mutations or the acquisition of exogenous genes. The acquisition of resistance genes frequently occurs by conjugation using pheromone-responsive plasmids, conjugative plasmids with a broad host range, or conjugative transposons with the potential to carry multiple antibiotic resistance genes [87,88]. Currently, this genus has revealed resistance to multiple antimicrobial drugs, such as β–lactams, aminoglycosides, amphenicols, fluoroquinolones, macrolides, tetracyclines, and glycopeptides (Table 1).

Enterococci express low-affinity penicillin-binding proteins (PBPs) that are responsible for their weak binding to β-lactam antibiotics [54]. *E. faecium* isolates from healthy poultry in Portugal have revealed a 30% rate of resistance to ampicillin [98]. Increased production of PBP5 has been associated with acquired resistance to penicillins (penicillin or ampicillin) among clinical *E. faecium* isolates [54,99]. In Portugal, *E. faecium* isolated from fecal samples of healthy broilers presented *pbp5* genes [89]. More recently, the *pbp5* gene was also identified in *E. hirae* isolated from apparently healthy broilers [90]. In *E. faecalis*, acquired ampicillin resistance is unusual but is mostly mediated by mutations in the *pbp*4 gene [100]. Hasan et al. (2018) reported high rates of *pbp4* genes in *E. faecalis* isolated from poultry environments (poultry feces, air, and feed) [59]. 

Low-level resistance to aminoglycosides, such as streptomycin or gentamicin, is the result of enterococci intrinsic resistance. However, the acquisition of aminoglycoside-modifying enzymes can lead to high-level resistance [87]. Enterococci isolated from broilers with vertebral osteomyelitis have shown high-level aminoglycoside resistance [101]. Kanamycin-resistant *E. faecalis* isolated from healthy broilers and *E. gallinarum* isolated from chicken meat contained the *aac*(6’)–*aph*(2”) gene [91,92]. This gene is also frequently responsible for gentamicin resistance in enterococci [102]. Gentamicin resistance has been found among enterococci isolated from humans, retail food, and healthy farm animals from six US states [103]. High levels of kanamycin resistance were also identified in *E. faecium* isolates from healthy poultry in Portugal [98].

Amphenicols are broad-spectrum antibiotics, and due to their toxicity and adverse effects in humans, chloramphenicol and its derivates were banned in 1994 from use in food-producing animals in the EU [104]. Accordingly, a low frequency of chloramphenicol resistance was observed among *E. faecium* and *E. faecalis* isolates from healthy broilers in Denmark [92,105]. More recently, a Turkish study performed with broilers from a slaughterhouse revealed a 33.1% rate of *Enterococcus* species resistant to chloramphenicol [106]. Chloramphenicol-resistant strains usually contain the *cat*_pIP501_ gene, and horizontal dissemination of phenicol resistance genes among enterococcal isolates may also contribute to the increase in chloramphenicol resistance [54,74]. Accordingly, *E. faecium* and *E. faecalis* isolates collected from healthy broilers in Denmark contained the *cat*_pIP501_ gene [92]. 

Linezolid, the first clinically available oxazolidinone, is globally used in human medicine as a last-resort antimicrobial agent to treat infections caused by multidrug-resistant Gram-positive pathogens, such as VRE [107,108]. This drug class is not approved for food animals in the USA and EU and, as expected, Tyson et al. (2018) reported very low levels of linezolid-resistant *Enterococcus* spp. (LRE) from food animal cecal content in the USA, and De Jong et al. (2019) revealed that commensal enterococci from healthy cattle, pigs, and chickens across Europe and broiler breeder farms in Korea were rarely resistant to linezolid [69,93,109]. In the United Arab Emirates, Habib et al. (2022) reported as well low levels of LRE from retail broiler meat [107]. Besides linezolid, daptomycin, which is a cyclic lipopeptide antibiotic, is also used for the treatment of complicated infections caused by Gram-positive organisms. Although there are no daptomycin formulations approved for animal use in the EU, Diarra et al. (2010) were able to isolate two daptomycin-resistant *Enterococcus* spp. (DRE) from broiler chickens [64,110]. The absence or very low levels of clinical resistance to several antibiotics that are highly valuable for human medicine, such as linezolid and daptomycin, is encouraging [69]. However, these findings add to the importance of monitoring the emergence of LRE and DRE at retail and farm levels. 

Cross-resistance to linezolid is attributed to different groups of acquired resistance genes [88]. Among them are the *cfr* gene, which confers transferable resistance to oxazolidinones, phenicols, lincosamides, pleuromutilins, and streptogramin A, and the *optrA* gene, which confers resistance to linezolid, tedizolid, chloramphenicol, and florfenicol [111,112]. Although not common among food animals, the *cfr* gene has been detected in *E. faecalis* isolated from retail chicken meat [113,114]. The *optrA* gene as well as the *fexA* gene were found among *E. faecalis* isolated from fecal samples of broilers [93]. A Chinese study reported that the *optr*A gene was more frequently detected in enterococci from food-producing animals (15.9%) than in humans (2.0%), which might suggest an animal reservoir or that the *optr*A gene has disseminated more quickly in enterococci of animal origin due to the selective pressure imposed by the use of florfenicol [111]. A high level of resistance against macrolide-lincosamide-streptogramin B (MLS_B_) has been shown by enterococci from the internal organs of healthy and diseased poultry. In addition, enterococci that express the *erm*B gene can also exhibit resistance to tetracycline [72,94]. Tetracycline-resistant *Enterococcus* isolates harboring *tetL*, *tetM*, *tetO*, or *tetS* in association with the *ermB* gene encoding resistance to MLS_B_ have been isolated from fecal and cloacal samples from broilers [64,94]. 

Vancomycin and teicoplanin are important members of the glycopeptides class, and resistance to vancomycin has been recently detected among 11% of the enterococci collected from cecal samples of healthy broilers at a Swedish slaughterhouse [54,115]. Glycopeptide resistance determinants have been detected in all farm species, and the mechanism of resistance usually involves altering the peptidoglycan synthesis pathway [54,116]. *E. faecium*, *E. faecalis*, and *E. hirae* isolated from broilers carried the *vanA* gene, as did VRE isolated from a Norwegian broiler production facility [49,96,97]. The *vanC* gene has been detected in *E. gallinarum* isolated from fecal and cecal samples of broilers [64]. 

### 3.3. Emergence and Dissemination of Vancomycin-Resistant Enterococci (VRE)

Enterococci were the first pathogens to show acquired resistance to vancomycin, and they emerged in the late 1970s as leading hospital-associated pathogens likely due to the extensive use of vancomycin to treat enterococcal infections [87,117,118]. The use of avoparcin as a growth promoter in farm animals may have also contributed to the emergence of vancomycin-resistant *Enterococcus* spp. (VRE) [39]. Moreover, in Europe, the VRE problem was initially confined to livestock, and VRE was observed in animals regularly exposed to antibiotics [92,97]. In the late 1990s, several food-producing animals, healthy humans, food products, and environmental samples, all over Europe and other countries, were colonized by VRE [49,91,95,97,98,119]. When avoparcin was banned as a growth promoter in the European Union, a decrease in VRE fecal carriage in animal meat products and human fecal flora was observed in a German study [120]. However, many reports suggested that VRE persisted in food animals. A Norwegian study documented a high prevalence of VRE in broiler and turkey carcasses three years after avoparcin was banned in Norway [49]. Denmark also presented similar findings [119]. In Sweden, the proportion of VRE-positive samples from healthy broilers increased from less than 1% in 2000 to over 40% in 2005 [96]. More recently, Leinweber et al. (2018) reported a 7.5% prevalence of vancomycin-resistant *E. faecium* in retail chicken meat [121]. Currently, VRE represents a serious threat to global health [122]. 

Resistance to vancomycin in enterococci has been mainly associated with the *vanA* and *vanB* gene clusters that allow the synthesis of different cell wall precursors with little affinity to vancomycin [110,123]. VRE containing the *vanA* gene are considered endemic, and they have been previously reported in human and animal samples, as well as in food and water [48,124,125,126]. Moreover, some enterococcal species, such as *E. gallinarum* and *E. casseliflavus,* have shown a different vancomycin resistance mechanism, related to a chromosomally encoded VanC operon [64,127]. All of these vast resistance characteristics limit therapeutic options, particularly the antibiotic treatment of nosocomial infections in humans and multiple diseases in poultry [56].

### 3.4. Molecular Characteristics of Enterococcus Clones

This review gathered information from studies that detected and characterized *Enterococcus* spp. from broilers or broiler meat. Table 2 and Table 3 present worldwide identified clonal lineages of *E. faecalis* and *E. faecium* isolated from broilers or broiler meat from 2018 to 2022. 

Stępień-Pyśniak et al. (2021) carried out a study that included 35 Polish *E. faecalis* and 41 Danish *E. faecalis* strains collected during post-mortem examination from broiler chicks showing lesions characteristic of yolk sac infection. The most prevalent clonal lineage among the Polish isolates was ST59, followed by ST282 and ST16. Regarding the Danish isolates, the most prevalent clonal lineages were ST116 and ST16. Only two Danish isolates were identified as VRE, and one belonged to the ST387 clonal lineage, while the other belonged to ST838 [128]. A Brazilian study that analyzed 12 *E. faecalis* strains isolated from natural cases of vertebral osteomyelitis in broilers revealed that almost half of these belonged to ST49. In addition, ST202 was represented by one strain that was vancomycin-resistant [101]. A study performed with 45 *E. faecalis* strains isolated from the cloaca of healthy broilers in Saudi Arabia reported that most of those strains belonged to ST16, ST302, and ST179, respectively. Two isolates were VRE, and these also belonged to ST16 [90]. In China, 61 strains of *E. faecalis* isolated from the cecal tissue of broiler chickens with swollen cecal lesions belonged to 34 sequence types, and the most prevalent was ST631 [129]. Kim et al. (2018) studied the molecular characteristics of 85 *E. faecalis* strains isolated from chicken meat samples, and ST256 was observed in over 50% of the isolates [130]. *E. faecalis* strains isolated from retail chicken carcasses in the Emirate of Abu Dhabi were assigned to five different sequence types, and half of them belonged to the clonal lineage ST476 [107]. ST314, followed by ST16, were the most prevalent clonal lineages reported among broilers across Australia [60]. Overall, according to the studies mentioned in Table 2, the most frequent and wide-ranging clonal lineage that has been identified among *E. faecalis* isolated from broilers or broiler meat since 2018 is ST16. This sequence type has already been identified in Poland, the Netherlands, Saudi Arabia, China, and Australia, and in both vancomycin-resistant and vancomycin-susceptible *E. faecalis*.

Leinweber et al. (2018) isolated three vancomycin-resistant *E. faecium* (VREfm) strains from Danish chicken meat, and all the strains belonged to ST32 [121]. VREfm strains were also isolated from cecal samples from healthy broilers in Sweden, but all of these belonged to ST310 [115]. In Turkey, a study that included vancomycin-susceptible *E. faecium* and VREfm isolated from broiler cloaca reported that ST1346 was the most prevalent clonal lineage among vancomycin-susceptible *E. faecium*, while all VREfm presented different and novel STs (ST1341, ST1342, ST1343, ST1244, and ST1345) [131]. A study that included 30 *E. faecium* strains isolated from the cloaca of healthy broilers in Saudi Arabia reported that most of those strains belonged to ST194, ST82, and ST157, respectively [90]. Kim et al. (2018) isolated one *E. faecium* strain from chicken meat samples that was revealed to belong to ST451 [130]. *E. faecium* isolated from retail chicken carcasses in Abu Dhabi Emirate has been assigned to four different sequence types: one known ST (ST195) and three novel STs (ST2236, ST2238, and ST2239) [107]. ST492, followed by ST195 and ST241, were the most prevalent clonal lineages reported among broilers across Australia [60]. Overall, according to the studies mentioned in Table 3, *E. faecium* isolates from broilers or broiler meat since 2018 do not share many clonal lineages. However, ST194 and ST195 were already identified in two different sources (broilers and broiler meat) on at least two different continents. 

## 4. *Escherichia coli*

*E. coli* are facultative, anaerobic Gram-negative rods that can be found in the intestinal tract of food-production animals and humans [132,133,134]. They are commonly acknowledged as antimicrobial resistance indicators in Gram-negative bacterial populations and are a model for antimicrobial resistance surveillance studies [23,134]. 

This bacterium has a special place in the microbiological world since it represents a substantial part of the endemic microbiota of different hosts and can also cause severe infections in humans and animals [135]. *E. coli* can be classified into different pathotypes capable of causing various diseases. Intestinal pathogenic *E. coli* (IPEC) are responsible for disorders in the gastrointestinal tract ranging from mild diarrhea to severe colitis [136,137,138]. In contrast, extraintestinal pathogenic *E. coli* (ExPEC) are mainly asymptomatic inhabitants of the intestinal tract that can cause extra-intestinal diseases after migrating to other body parts, such as the urinary tract or the bloodstream [139,140]. ExPEC has already been isolated from healthy production chickens in Canada, from diseased broiler chickens in Egypt, and from meat chickens in Australia [141,142,143].

Avian pathogenic *E. coli* (APEC), a subset of ExPEC, is mainly responsible for respiratory or systemic infections in poultry [144,145]. Additionally, in poultry production, it is a major cause of colibacillosis, which is considered the main cause of decreased productivity and increased mortality, leading to major economic losses [146,147,148]. Colibacillosis is characterized by acute fatal septicemia or sub-acute fibrinous pericarditis, airsacculitis, salpingitis, and peritonitis [149]. Good husbandry, strict biosecurity, and vaccination are essential to prevent colibacillosis. Vaccination against colibacillosis is generating interest, and Śmiałek et al. (2020) have already reported that vaccination decreased the number of *E. coli* isolates from broilers of commercial farms and that these isolates were more susceptible to the antimicrobials [150]. Ebrahimi-Nik et al. (2018) also showed an efficient vaccine against colibacillosis in poultry [151]. However, since different strains can cause outbreaks, it is challenging to develop a vaccine that is effective against multiple strains [152]. 

### 4.1. Antimicrobial Resistance in E. coli 

In recent decades, a growing number of resistance genes have been identified in *E. coli* isolates, and many of these were acquired by horizontal gene transfer. *E. coli* can act as a donor or a recipient of resistance genes, so resistance genes can be passed on or acquired by *E. coli* [135]. Several studies have reported that both commensal and pathogenic *E. coli* are prevalent in broiler chickens and that most of the isolates revealed resistance to ampicillin, tetracycline, ciprofloxacin, nalidixic acid, and sulfamethoxazole-trimethoprim (Table 4). 

Ampicillin is a β-lactam antibiotic, and resistance to β-lactams in Gram-negative bacteria is primarily mediated by β-lactamase enzymes that hydrolyze the β-lactam ring, thereby inactivating the drug [145]. A study performed with cloacal swab samples from apparently healthy broilers revealed that the *E. coli* isolates were 100% resistant to ampicillin [160]. Ampicillin-resistant *E. coli* were screened for several genes, and the most frequently found were *bla*_TEM_, *bla*_SHV_, *bla*_OXA_, *bla*_CMY_, and *bla*_CTX-M_ [153,154]. Al Azad et al. (2019) and Sarker et al. (2019) both revealed a high prevalence of *bla*_TEM_ in *E. coli* isolated from cloacal swabs of broiler chickens [160,161]. The genes *bla*_CTX,_ *bla*_CMY,_ and *bla*_SHV_ have also been identified in *E. coli* isolated from broilers [154,162].

Tetracyclines are among the most common therapeutic agents used in animals. A. M. Ahmed et al. (2013), reported that 91.8% of the APEC isolates from septicemic broilers in Egypt harbored tetracycline resistance determinants [145]. Tetracycline resistance in broilers is possibly due to the acquisition of the *tet*A gene [155].

A study that included cecal samples from healthy broilers and broiler meat revealed a high prevalence of antimicrobial resistance, particularly quinolone resistance [163]. Regarding the quinolone-resistant genes, *qnr*A and *qnr*S play an important role in broiler chickens [156,157]. De Koster et al. (2021) reported resistance to ciprofloxacin in *E. coli* isolated from Belgian and Dutch broiler farms [164]. Mutations in the *gyrA* and *gyrB* genes could be the molecular mechanisms responsible for the acquisition of ciprofloxacin resistance [155]. Resistance of *E. coli* from broiler breeding animals, that had just arrived in Sweden, to nalidixic acid was identified by Börjesson et al. (2016), suggesting that the importation of birds can be a source of the occurrence of these bacteria in Swedish broiler production [165]. 

Sulfonamides are listed for use in poultry in all countries and, according to Roth et al. (2019), the resistance rates in *E. coli* of broiler origin to sulfamethoxazole are higher than 40% in all countries [23]. One Portuguese research study focused on the resistance of *E. coli* isolated from carcasses and internal organs of healthy chickens from intensive farms detected *sul1* as the most common gene of the sulfonamide class [166]. On the other hand, the *sul2* gene was the most prevalent gene detected in isolates from broilers, Danish broiler meat, and imported broiler meat [159]. However, when analyzing the genes involved in sulfamethoxazole-trimethoprim resistance, it is necessary to consider the genes *sul* and *dfr*, since they act synergistically to confer resistance [167]. Genes *dfrA1*, *dfrA12*, *dfrA14,* and *dfrA*17 were the most commonly identified genes in trimethoprim-resistant strains of avian fecal *E. coli* recovered from clinically healthy chickens [158]. 

### 4.2. ESBL-Producing E. coli 

As a member of the *Enterobacteriaceae* family, *E. coli* can produce extended-spectrum β-lactamases (ESBLs) [168,169]. ESBLs are enzymes that can degrade extended-spectrum β-lactam antibiotics, such as third-generation cephalosporins, commonly used to treat numerous systemic infections [170]. Organisms capable of producing ESBLs were first reported in Europe in the early 1980s, and since then, their prevalence rates increased [171,172]. ESBL-producing *E. coli* are becoming the most challenging multidrug-resistant pathogens worldwide, and they have been extensively described among broilers [133,154,173,174,175]. In particular, Rousham et al. (2021) reported a high prevalence of ESBL-producing *E. coli* in broiler ceca and feces in households, farms, and live poultry. Furthermore, this study also revealed that the majority of the isolates were resistant to fluoroquinolones, cefepime, sulfonamides, and aminoglycosides [42]. Interestingly, a study performed by Van Hoek et al. (2018) showed that almost 30% of one-day-old broilers were already ESBL-positive [44].

ESBLs can be categorized into three main subtypes: TEM, SHV, and CTX-M β-lactamases. The TEM and SHV subtypes are large and widespread groups that differ from their parental enzymes by one or two amino acids [176]. However, these minor alterations in their amino acid sequences are sufficient to extend the spectrum of their enzymatic activity, which allows them to hydrolyze cephalosporins that have an oxyimino side chain, such as third-generation cephalosporins and aztreonam [177]. Both TEM and SHV subtypes were reported in the United States and France in the late 1980s and 1990s [169,178]. Unlike other ESBLs, the CTX-M family is a heterogenous and complex group of enzymes that possibly resulted from the relocation of chromosomal Kluyvera genes and that can confer resistance to cefotaxime and ceftazidime [179].

TEM and SHV types were the prevailing ESBL enzymes worldwide for a long time. Now, CTX-M enzymes may represent the most prevalent subtype of ESBLs [180,181,182]. Worryingly, a significant proportion of ESBL-producing isolates are represented by *E. coli*-expressing CTX-M β-lactamases that have quickly spread not only among healthcare settings but also in the community [42,139]. The spread of CTX-M variants in animals and humans is responsible for the high frequency of ESBLs [132,183,184]. An Indonesian study revealed a prevalence of almost 97.8% of CTX-M-producing *E. coli* among broilers’ cloacal swabs [174]. Currently, over 123 types of CTX-M have been identified [139].

CTX-M-14 and CTX-M-15 are extensively disseminated among chickens [185,186,187]. Still, the CTX-M-1 gene has also been reported as one of the common CTX-M types that have been recovered from poultry in many European countries [140,154,188,189]. Liu et al. (2020) revealed that CTX-M-14 was the most predominant CTX-M subtype identified among apparently healthy broiler chickens, and CTX-M-14 has also been detected in broiler meat in Portugal [190,191]. Subramanya et al. (2020) collected samples from healthy poultry from backyard farms and commercial broiler farms, and their data indicated that CTX-M-15 was the most prevalent ESBL enzyme [192]. CTX-M-15-producing *E. coli* is strongly linked to sequence type 131 (ST131) clones, which are related to fluoroquinolone resistance [168,193]. Many European countries use fluoroquinolones in farm animals that could be related to the fluoroquinolone-resistant *E. coli* strains [194]. Moreover, fluoroquinolones are approved for use in poultry in the largest poultry-producing countries, with the exception of the US [23,195]. However, a study from Awawdeh et al. (2022) reported fluoroquinolone-resistant *E. coli* from meat chickens in Australia, a country that does not use fluoroquinolones in poultry, which suggests that this resistance is likely due to horizontal transmission of antibiotic-resistant genes [143,196]. The CTX-M-1 gene was described in commensal isolates from French layers and healthy poultry [188,197]. Moreover, a Dutch study that collected samples from an organic broiler farm revealed that all *E. coli* isolates carried CTX-M-1 genes [44]. 

### 4.3. Molecular Characteristics of E. coli Clones

This review article gathered information from studies that detected and characterized *E. coli* from broilers or broiler meat. Table 5 shows worldwide identified clonal lineages of *E. coli* isolated from broilers or broiler meat from 2020 to 2022. 

Päivärinta et al. (2020) collected broiler cecal samples from a high-capacity slaughterhouse and from vacuum-packed raw broiler meat without marinade intended for consumer use, all from the same high-capacity slaughterhouse. In total, three ESBL-producing *E. coli* strains were isolated: two from the ceca that belonged to ST1594, and one from the meat that belonged to ST351 [175]. Retail chicken meat was also studied in Egypt, and ST1196 was the most prevalent sequence type among ESBL-producing *E. coli*, while ST156 and ST189 were identified among non-ESBL-producing *E.coli* [203]. Broilers infected with colibacillosis were studied in Norway, Croatia, Tunisia, and Pakistan [144,147,198,199]. In the Norwegian study, ST429 accounted for over 60% of the clonal lineages identified in *E. coli* isolates [144]. However, in Croatia, ST429 was reported at a much lower rate (0.65%). The most prevalent sequence types in Croatia were ST95 and ST117 [198]. ST117 was also predominant among the Pakistani *E. coli* isolates from broilers with colibacillosis [201]. The Tunisian study reported four different sequence types in ESBL-producing *E. coli* strains, with the majority belonging to ST4187 [147]. Two different Pakistani studies that included cecal and fecal samples from broilers reported ST131 between the most prevalent sequence types in ESBL-producing *E. coli* strains [200,202]. A study carried out by Aslantaş (2020) in Turkey detected 19 sequence types in 28 ESBL-producing *E. coli* isolates, and the most prevalent were ST114 and ST354 [199]. In Australia, ESBL-producing *E. coli* isolated from healthy broilers belonged to different clonal lineages, while *E. coli* from chickens with colibacillosis belonged mainly to ST354 [143]. Overall, according to the studies mentioned in Table 5, the most frequent and wide-ranging clonal lineage that was identified in both ESBL-producing and non-ESBL-producing *E. coli* isolated from broilers or broiler meat since 2020 was ST117.

## 5. Impact of Antibiotic Usage and Antibiotic-Resistant Bacteria in the Gastrointestinal Tract of Broilers: A One Health Approach

The use and abuse of antibiotics select and enrich antibiotic-resistant bacteria in the gastrointestinal microbiota of food animals, particularly broilers [204]. Therefore, antibiotic-resistant bacteria may be carried in the large intestine of adult laying hens and shed in their feces, leading to contamination of the eggshell surface [3]. Specific foodborne and poultry pathogens found on the eggshell surface might infect the hatchlings and consequently affect the health of the growing broiler and their derived meat products [205]. The antimicrobial-resistant bacteria that have emerged and live in the animal production environment can spread to humans through human–animal contact or the consumption of or contact with animal products [121,122,206]. Furthermore, during food processing, when an animal is slaughtered, the muscles are exposed and can be cross-contaminated if the gastrointestinal tract ruptures or if contaminated instruments and materials are used [207]. An American study reported that 95% of retail chicken meat samples were contaminated with enterococci, mainly with *E. faecium*, followed by *E. faecalis* [57]. Adeyanju and Ishola (2014) revealed that almost 44% of retail chicken samples from Nigeria presented *E. coli* [208].

**Figure 2:**
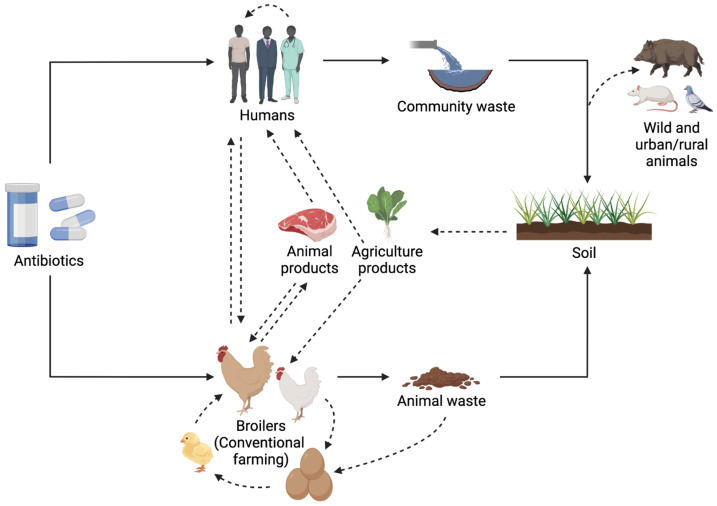
Spread of antibiotic residues and antimicrobial resistance according to a One Health approach. Modified from [214]. Information extracted from [13,121,122,167,206,211,213,215,216].

Antibiotic usage is the most important factor that provides the selection pressure that enables the dissemination of antimicrobial resistance genes, and unfortunately, antibiotic exposure is not only caused by antibiotic consumption [209,210]. Almost 90% of the administered doses of antibiotics are excreted unmodified or partly metabolized through urine and feces [25]. Animal manure is acknowledged as a rich reservoir of antibiotic residues, and its use as crop fertilizer exposes the environment to antibiotic determinants [13]. Antibiotic-resistance genes can persist in soils for several weeks, and their transmission to crops, and therefore, to animals or humans when consumed, represents a health risk [211]. Discharges from the wastewater treatment process also represent a way for resistant bacteria to enter the environment [212]. Once in the environment, the bacterial resistance can be transferred to wild animals, such as wild birds, particularly migratory raptors, who travel long distances through different ecological niches and prey on synanthropic rodents and small birds in urban and rural environments (Figure 2) [122,213].

Recently, broilers have increased significantly as a meat source, and the largest broiler meat producers worldwide include the United States, China, and Brazil, respectively. Within the European Union, Poland, Spain, Germany, France, and Italy present the higher gross domestic production of broilers [217]. Broiler meat produced by some of these countries is exported globally [23]. For example, Brazil is the world’s largest poultry exporter; about a third of Brazil’s chicken production is exported—4.6 million out of 14.3 million metric tons in 2020—to over 150 countries worldwide [218]. Therefore, ongoing surveillance systems for antimicrobial resistance in broiler production are mandatory to avoid the spread of antimicrobial resistance among broiler meat or other foods derived from these animals. 

### 5.1. Transmission of Enterococcus spp. and E. coli 

Some enterococci, especially *E. faecalis* and *E. faecium*, have been increasingly associated with hospital-acquired infections in human and veterinary medicine [219]. From 2010 to 2020, 6.1% to 17.5% of strains isolated from European human patients with hospital-acquired infections were reported to be enterococci [220]. A Danish review suggested that *E. faecium* isolates of animal origin might not constitute a human health hazard, but could act as donors of antimicrobial resistance genes for other pathogenic enterococci [221]. Human infections can also be caused by *E. hirae*, and these mostly involve bacteremia accompanied by severe illness, such as acute pyelonephritis, pancreatitis, cholangitis, severe urinary tract infections, or spondylodiscitis [71]. Fortunately, human endocarditis caused by *E. hirae* has been hardly described [73,222,223]. Reports on human infections caused by *E. cecorum* are extremely rare [76,224,225,226,227]. However, *E. cecorum* strains were found in broiler breeders or broiler chickens, and they are thought to be a source of transmission leading to *E. cecorum*-associated septicemia in humans [75,77]. Domestic animals such as cats and dogs are also possible sources of transmission [75,76,77]. Chickens have been described as VRE reservoirs [49,119,121,219]. Vancomycin-resistant *E. faecium* isolated from chickens in Malaysia revealed similarities to those from humans. However, the unusual detection of human enterococci clones in chickens may suggest a reverse transmission of enterococci from humans to animals [219]. 

Foodborne *Enterococcus* spp. are rarely considered pathogens, but consumption of these bacteria enables their establishment in the gastrointestinal tract [228]. In addition, the presence of antimicrobial resistance genes in *Enterococcus* species, mainly those on mobile elements, allows the transfer of these genes to other gastrointestinal bacteria [92,94]. Due to their ability to survive gastric passage and multiply, resistant *E. faecium* bacteria isolated from chicken meat were detected in feces for up to 14 days after ingestion [228]. Furthermore, these enterococci might be able to cause many diseases, representing a public health hazard [130]. The presence of multidrug-resistant enterococci has been detected worldwide, including in healthy broilers from Canada, Greece, Italy, and Poland [64,206,229,230]. 

*E. coli*, like other gastrointestinal bacteria, can form a reservoir of antibiotic-resistant genes capable of causing disease in both humans and animals [23]. Since *E. coli* can cause life-threatening infections, the transmission of virulent and resistant *E. coli* among animals and humans through direct contact, contact with animal excretions, or via the food chain is a major concern [135,231]. A study performed in Iceland reported that resistant *E. coli* bacteria isolated from feed, broilers, broiler meat, and humans were closely related, revealing that poultry and their food products can be a source of resistant *E. coli* to humans [163]. Resistant *E. coli* can also transmit their resistance genes to different bacterial species that can cause many diseases in both humans and animals [23,231,232]. 

Several studies have reported antimicrobial resistance in poultry, but only a few have investigated the breeders [133,153,154,233]. However, a study by Noh et al. (2020) reported *E. faecalis* isolated from broiler breeders and resistant to a diverse range of antimicrobials, implying their potential role as reservoirs for the transmission of resistant isolates throughout the poultry industry [234]. Furthermore, ExPEC isolates were found among diseased broilers and healthy chickens [141,142,235]. Retail meats are also frequently contaminated with ExPEC strains. Researchers found that human and animal-source ExPEC shared highly similar virulence genes and clonal backgrounds, indicating that chicken meat has been a source of ExPEC to humans [236,237]. Stromberg et al. (2017) also revealed that fecal ExPEC can contaminate chicken carcasses at slaughter and then spread to humans via animal product consumption or direct contact [141].

ESBL-producing bacteria might also be transmitted from human to human or from animal to human via direct contact or the food chain. Falgenhauer et al. (2019) found three very closely related broiler/human isolate clusters, implying that poultry farms or meat products are important sources of ESBL-producing bacteria [238]. Furthermore, the high prevalence of CTX-M-15 *E. coli* revealed in a Romanian broiler production chain adds importance to the role that chickens play as a reservoir of resistance genes for humans [187]. The impact of infections caused by ESBL-producing *E. coli* in farm animals is still unpredictable. Nevertheless, to keep this threat under control, the animals’ potential as reservoirs for these bacteria needs to be assessed from a One Health perspective [239]. 

Daniel et al. (2017) reported a high prevalence of multidrug-resistant *E. faecalis*, including vancomycin resistance, in river water, closely followed by wastewater, while different Malaysian studies isolated VRE from poultry drinking water, implicating VRE stabilization in the environment [61,240]. Päivärinta et al. (2020) studied the prevalence of ESBL-producing *E. coli* in different broiler flocks, farms, and broiler meat from Finland, where there is no use of antibiotics, and the results revealed that 18% of cloacal samples and 32% of meat samples presented ESBL-producing *E. coli* [175]. Retail chicken meat and chicken samples from antibiotic-free and organic farms also revealed resistant *E. coli* strains [241,242]. This evidence suggests that other potential infection sources, such as river water, feed, or vectors, are very important in the transmission epidemiology of VRE and ESBL-producing *E. coli*. 

### 5.2. Clonal Relationship from a One Health Perspective

*E. faecalis* ST16 isolated from yolk sac infections was previously characterized as an epidemic clone in hospitals in Poland and other European countries [243,244]. Its presence has also been detected in many animals, such as poultry, pigs, and cattle [245]. *vanA E. faecalis* ST116 isolates were isolated from turkey meat, non-hospitalized humans, and patients [123]. *E. faecalis* sequence types ST4, ST59, ST82, ST116, and ST245 have been found in hospitalized patients [245]. ST49 was detected more frequently in hospitalized human patients than in non-hospitalized human patients [246]. Furthermore, ST16, ST21, ST179, and ST480 have been reported among *E. faecalis* hospital isolates in Saudi Arabia [247]. In China, human ST631 derived from *E. faecalis* primarily manifests in diseases of the abdominal cavity, gastrointestinal tract, and other related sites [129]. ST256 has been isolated from chickens, pigs, and humans and has shown a high prevalence of multidrug resistance [130,248,249]. Furthermore, *E. faecalis* ST256 carries the *optrA* gene, which is related to oxazolidinone and phenicol resistance [111,248]. Freitas et al. (2020) reported genetic relatedness between *optrA*-positive *E. faecalis* of ST476 in animal and clinical (human) hosts worldwide over several decades [250]. 

Alzahrani et al. (2022), described *E. faecium* isolates from eight different sequence types in cloacal swabs from healthy broilers. Five of these belonged to CC9 (ST9, ST157, ST82, ST194, and ST12) and three to CC17 (ST16, ST18, and ST360) [90]. CC17 was considered a nosocomial clonal complex, but several studies have reported the dissemination of *E. faecium* CC17 in animals [123,219]. Chickens can possibly acquire the CC17 *E. faecium* isolates from contaminated environments or humans visiting the farm. This idea is reinforced by a study that showed the transmission of *E. faecium* of human origin to chickens [251]. *E. faecium* ST492 isolates found in broilers were clustered with the human isolates, which may also indicate reverse zoonotic transmission from humans to chickens along the production chain [60].

Two cecal *E. coli* ST1594 isolates that came from two different flocks from the same farm carried *bla*_CTX-M-1_ and *sul2* resistance genes. These findings indicate that clonal transfer of *bla*_CTX-M-1_ and *sul2* genes may occur between different *E. coli* ST1594 strains [175]. MLST findings by Ramadan et al. (2020) showed overlapping *E. coli* STs from different sources: ST1011, ST156, ST48, and ST224 in chicken and beef isolates; ST10 in human and chicken; and ST226 in human and beef isolates. This suggests the adaptability of some STs to distinctive hosts with a potential for inter-species transmission [203]. Furthermore, ST10 and ST48 belonged to CC10, which is linked to diarrheagenic *E. coli* infections in humans worldwide [252]. *E. coli* ST429 has been identified as APEC [253]. ST95 has also been previously associated with APEC. In addition, uropathogenic *E. coli* ST95 has been isolated from humans [254]. In the study carried out by Kravik et al. (2018), both ST429 and ST95 were analyzed to deduce their phylogenetic relationship, and ST429 revealed a high sequence resemblance between isolates from the same flock, while ST95 isolates from a single flock were more diverse [144]. ST69, ST23, and ST131 are also frequently responsible for extraintestinal infections in humans and poultry [255,256,257]. As a carrier of many resistance and virulence-associated genes, ST131 has been often described as the accountable agent for human urinary infections and bacteremia [258]. ST131 is considered a well-established pandemic clone, and it was isolated from poultry samples from different European countries [132]. Jouini et al. (2021) isolated the pandemic high-risk human lineage CTX-M-15-B2-O25b-ST131 *E. coli* from diseased chickens in Tunisia [136]. ST117 was already reported in several Nordic countries, as well as in Canada [186,259]. The ST4187 lineage has been considered relevant regarding the spread of *mcr-1*-mediated colistin resistance and ESBL-encoding genes in *E. coli* isolates from broilers with colibacillosis [147]. ST4187 was also described in *E. coli* isolated from hospitalization units in Angola and birds from Chile [260,261]. 

## 6. Conclusions

Antimicrobials have played an essential role in diminishing mortality and morbidity rates in animal production. However, their misuse is considered one of the major threats to public health. The inappropriate application of antibiotics contributed to the selection and enrichment of antibiotic-resistant bacteria in the gastrointestinal microbiota of animals, and the consumption of contaminated animal-source food, direct contact with animals, or environmental exposure can lead to the transmission of antimicrobial-resistant bacteria to humans. In addition, food-animal waste may contain antimicrobial residues that will lead to the contamination of the environment, and consequently, to the spread of antimicrobial resistance to other sources.

The results presented in this review cannot exclude the possibility that pathotypes of *Enterococcus* spp. and *E. coli* isolated from broilers might represent transmission to or from humans. Infections by antibiotic-resistant bacteria are an increasing problem, and antimicrobial resistance can be responsible for treatment failures for both animal and human diseases, which have significant economic and public health consequences, such as prolonged treatment and extended hospital stays, which might further promote the transmission of resistant pathogens in hospitals and represent a financial burden. Furthermore, results concerning foodborne strains suggest that the food chain also represents a possible means of bacterial infection in humans. 

Therefore, the inappropriate use of antimicrobials in broiler chicken production is a primary concern, and it is imperative to restrict the use of critically important antibiotics for humans in food animals and explore antibiotic alternatives for animal production. Practices to prevent bacterial cross-contamination and manure treatment options that avoid the dissemination of antibiotic resistance into the environment are also important. In addition, to prevent and control the spread of antibiotic resistance, individuals should only use antibiotics when and as prescribed by a certified health professional, never share or use leftover antibiotics, prevent infections by regularly washing hands, avoiding close contact with sick people, and keeping vaccinations up to date, prepare food hygienically, and choose foods that have been produced without the use of antibiotics for growth promotion or disease prevention in healthy animals. To better understand the potential of antimicrobial resistance transmission, more studies regarding human and veterinary epidemiology are needed. 

## Figures and Tables

**Table 1:** Common resistant genes in enterococcus species isolated from broilers or broiler meat.

Class of Antibiotics	Resistance Genes	Enterococcus Species	Source	Ref.
β-lactams	*pbp*5	*E. faecalis* *E. faecium*	Fecal samples from healthy broilers	[89]
		*E. hirae*	Cloacal samples from healthy broilers	[90]
Aminoglycosides	*aac*(6’)–*aph*(2”)	*E. faecalis* *E. faecium*	Fecal samples from healthy broilers	[89]
		*E. gallinarum*	Retail chicken meat	[91]
Amphenicols	*cat*_pIP501_	*E. faecalis* *E. faecium*	Fecal samples from healthy broilers	[92]
Oxazolidinones	*optrA*	*E. faecalis* *E. faecium*	Fecal samples from broilers	[93]
	*fexA*			
Macrolides	*ermB*	*E. faecalis* *E. faecium*	Fecal samples from healthy broilers	[89]
		*E. hirae*	Cloacal samples from healthy broilers	[90]
Tetracyclines	*tetL*	*E. faecalis* *E. faecium* *E. hirae* *E. gallinarum*	Fecal and cecal samples from broilers	[64]
	*tetM*	*E. faecalis* *E. faecium* *E. hirae* *E. gallinarum* *E. casseliflavus* *E. durans*	Cecal samples from broilers	[94]
	*tetO*	*E. faecalis* *E. faecium*	Fecal samples from healthy broilers	[92]
		*E. hirae*	Fecal and cecal samples from broilers	[64]
	*tetS*	*E. gallinarum* *E. casseliflavus*	Cecal samples from broilers	[94]
Glycopeptides	*vanA*	*E. faecalis*	Fecal samples from broilers	[95]
		*E. faecium*	Cecal samples from healthy broilers	[96]
		*E. hirae*	Fecal samples from broilers	[97]
	*vanC*	*E. gallinarum*	Fecal and cecal samples from broilers	[64]

**Table 2:** Clonal lineages identified in *E. faecalis* isolated from broilers or broiler meat.

Country	Source	VRE	Clonal Lineages (Number and Prevalence of Isolates)	Ref.
Poland	Yolk sac from infected broilers	-	ST59 (11/35, 31.43%) ST282 (6/35, 17.14%) ST16 (3/35, 8.57%) ST36, ST82, ST836 (2/35, 5.71% each) ST65, ST93, ST116, ST165, ST302, ST529, ST837, ST840, ST843 (1/35, 2.86% each)	[128]
Netherlands	Yolk sac from infected broilers	-	ST116 (7/39, 17.95%) ST16 (6/39, 15.38%) ST36, ST82, ST245 (3/39, 7.69% each) ST4, ST100 (2/39, 5.13% each) ST32, ST49, ST59, ST65, ST202, ST282, ST302, ST363, ST387, ST529, ST839, ST841, ST842, ST844 (1/39, 2.56% each)	[128]
		+	ST387, ST838 (1/2, 50% each)	
Brazil	Vertebral osteomyelitis lesions from infected broilers	-	ST49 (5/11, 45.45%) ST100, ST116, ST249, ST300, ST708, ST709 (1/11, 9.09% each)	[101]
		+	ST202 (1/1, 100%)	
Saudi Arabia	Cloaca from healthy broilers	-	ST302 (8/43, 18.60%) ST179 (6/43, 13.95%) ST41, ST480 (5/43, 11.63% each) ST21, ST752 (3/43, 6.98% each) ST176 (2/43, 4.65%) ST32, ST81, ST177 (1/43, 2.33% each)	[90]
		+	ST16 (2/2, 100%)	
China	Ceca from broilers with cecal enlargement	ND	ST631 (7/61, 11.48%) ST634 (5/61, 8.20%) ST4, ST480, ST758 (4/61, 6.56% each) ST32, ST195 (3/61, 4.92% each) ST10, ST257, ST314, ST363, ST968 (2/61, 3.28% each) ST16, ST33, ST38, ST49, ST69, ST80, ST143, ST169, ST198, ST251, ST256, ST262, ST265, ST452, ST476, ST479, ST650, ST689, ST736, ST862, ST991 (1/61, 1.64% each)	[129]
Korea	Retail chicken meat	-	ST256 (44/85, 51.76%) ST32 (7/85, 8.24%) ST21, ST27, ST538 (5/85, 5.88% each) ST36 (4/85, 4.71%) ST833 (3/85, 3.53%) ST476, ST834 (2/85, 2.35% each) ST82, ST86, ST93, ST309, ST445, ST662, ST729, ST835 (1/85, 1.18% each)	[130]
United Arab Emirates	Retail chicken meat	-	ST476 (5/10, 50%) ST1184 (2/10, 20%) ST314, ST1290, ST1291 (1/10, 10% each)	[107]
Australia	Ceca from broilers	-	ST314 (7/37, 18.92%) ST16 (5/37, 13.51%) ST502, ST530 (4/37, 10.81% each) ST202, ST444, ST835 (2/37, 5.41% each) ST22, ST59, ST82, ST100, ST136, ST249, ST287, ST403, ST477, ST616, ST634 (1/37, 2.70% each)	[60]

VRE: vancomycin-resistant enterococci; -: VRE-negative, +: VRE-positive; ND: not described.

**Table 3:** Clonal lineages identified in *E. faecium* isolated from broilers or broiler meat.

Country	Source	VRE	Clonal Lineages (Number and Prevalence of Isolates)	Ref.
Denmark	Retail chicken meat	+	ST32 (3/3, 100%)	[121]
Sweden	Ceca from healthy broilers	+	ST310 (11/11, 100%)	[115]
Turkey	Cloaca from broilers	-	ST1346 (7/11, 63.64%) ST1348 (3/11, 18.18%) ST1347, ST1354 (1/11, 9.09% each)	[131]
		+	ST1341, ST1342, ST1343, ST1244, ST1345 (1/5, 20% each)	
Saudi Arabia	Cloaca from healthy broilers	-	ST194 (8/30, 26.67%) ST82, ST157 (5/30, 16.67% each) ST9 (4/30, 13.33%) ST16 (3/30, 10.00%) ST18, ST360 (2/30, 6.67% each) ST12 (1/30, 0.33%)	[90]
Korea	Retail chicken meat	-	ST451 (1/1, 100%)	[130]
United Arab Emirates	Retail chicken meat	-	ST2236 (3/6, 50%) ST195, ST2238, ST2239 (1/6, 16.67% each)	[107]
Australia	Ceca from broilers	-	ST492 (7/46, 15.22%) ST195, ST241 (5/46, 10.87% each) ST124 (4/46, 8.70%) ST10, ST507, ST517, ST640 (3/46, 6.52% each) ST8, ST236, ST1243 (2/46, 4.35% each) ST158, ST190, ST194, ST240, ST245, ST511, ST944 (1/46, 2.17% each)	[60]

VRE: vancomycin-resistant enterococci; -: VRE-negative; +: VRE-positive.

**Table 4:** Common resistant genes in *E. coli* isolated from broilers or broiler meat.

Class of Antibiotics	Resistance Genes	Source	Ref.
β-lactams	*bla*_TEM_	Cloacal samples from broilers	[153]
	*bla*_SHV_		
	*bla*_CTX_		
	*bla*_CMY_	Fecal samples from healthy broilers	[154]
Tetracyclines	*tet*A	Cloacal samples from broilers	[155]
Quinolones	*qnr*A	Liver samples from broilers infected with colibacillosis	[156]
	*qnr*S	Cloacal samples from healthy broilers	[157]
Sulfonamides	*dfrA*	Fecal samples from healthy broilers	[158]
	*sul2*	Fecal samples from broilers and broiler meat	[159]

**Table 5:** Clonal lineages identified in *E. coli* isolated from broilers or broiler meat.

Country	Source	ESBL	Clonal Lineages (Number and Prevalence of Isolates)	Ref.
Finland	Retail chicken meat	+	ST351 (1/1, 100%)	[175]
	Ceca from broilers	+	ST1594 (2/2, 100%)	
Norway	Colibacillosis lesions from infected broilers	ND	ST429 (29/47, 61.70%) ST95 (8/47, 17.02%) ST10836 (4/47, 8.51%) ST457 (3/47, 6.38%) ST40, ST2485, ST6665 (1/47, 2.13% each)	[144]
Croatia	Colibacillosis lesions from infected broilers	ND	ST95 (26/154, 16.88%) ST117 (23/154, 14.94%) ST390 (12/154, 7.79%) ST23 (11/154, 7.14%) ST162 (7/154, 4.55%) ST10, ST131 (6/154, 3.90% each) ST48 (4/154, 2.60%) ST135 (3/154, 1.95%) ST93, ST428 (2/154, 1.30% each) ST46, ST58, ST69, ST101, ST297, ST429, ST616, ST746, ST1485, ST3232, ST7013, ST8573 (1/154, 0.65% each)	[198]
Turkey	Cloaca from broilers	+	ST114, ST354 (3/28, 10.71% each) ST156, ST157, ST174, ST362, ST5114, ST5696 (2/28, 7.14% each) ST10, ST95, ST457, ST539, ST648, ST1158, ST1640, ST4248, ST5843, ST6635 (1/28, 3.57% each)	[199]
Pakistan	Ceca from broilers	+	ST131 (22/48, 45.83%) ST8051 (10/48, 20.83%) ST2847, ST8900 (2/48, 4.17% each) ST2741, ST3499, ST6293, ST8420, ST8431 (1/48, 2.08% each)	[200]
	Colibacillosis lesions from infected broilers	+	ST117 (10/28, 35.71%) ST2847 (8/28, 28.57%) ST23, ST48 (3/28, 10.71% each) ST69 (2/28, 7.14%) ST101, ST350, ST602, ST1011, ST5704 (1/28, 3.57% each)	[201]
		-	ST117 (2/6, 33.33%) ST10, ST48, ST162, ST752, ST1727 (1/6, 16.67% each)	
	Feces from broilers	+	ST1035 (11/26, 42.31%) ST131 (8/26, 30.77%) ST1215 (5/26, 19.23%) ST2279 (2/26, 7.69%)	[202]
		-	ST1650 (3/9, 33.33%) ST188 (2/9, 22.22%) ST110, ST123, ST410, ST3059 (1/9, 11.11% each)	
Tunisia	Colibacillosis lesions from infected broilers	+	ST4187 (4/7, 57.14%) ST3882, ST5693, ST8932 (1/7, 1.43% each)	[147]
Egypt	Retail chicken meat	+	ST1196 (7/59, 11.86%) ST162 (6/59, 10.17%) ST189, ST69, ST117, ST1011 (4/59, 6.78% each) ST93, ST8594 (3/59, 5.08% each) ST10, ST155, ST206, ST224, ST608, ST744 (2/59, 3.39% each) ST48, ST57, ST155, ST212, ST302, ST359, ST457, ST997, ST1011, ST1072, ST1684, ST2179 (1/59, 1.69% each)	[203]
		-	ST156, ST189 (1/2, 50% each)	
Australia	Cloaca from healthy broilers	+	ST10, ST224, ST624 (1/3, 33.33% each)	[143]
	Colibacillosis lesions from infected broilers	-	ST354 (4/7, 57.14%) ST57, ST2705, ST6053 (1/7, 14.29% each)	

ESBL: extended-spectrum beta-lactamase; -: ESBL-negative; +: ESBL-positive; ND: not described.

## Data Availability

No new data were created or analyzed in this study. Data sharing is not applicable to this article.
